# From Isolation to Pilot-Scale Production: *Enterococcus faecium* YC07 with Urate-Lowering Potential from Fermented Food Jiangshui

**DOI:** 10.3390/foods14122076

**Published:** 2025-06-12

**Authors:** Xiaoyu Cao, Qianqian Xu, Yu Zhang, Hai Yan

**Affiliations:** School of Chemistry and Biological Engineering, University of Science and Technology Beijing, Beijing 100083, China; d202210452@xs.ustb.edu.cn (X.C.);

**Keywords:** *Enterococcus faecium* YC07, whole genome sequencing, phenotypic analysis, pilot-scale fermentation, urate-lowering potential

## Abstract

Hyperuricemia arises from urate overproduction and/or underexcretion. Probiotics offer the potential for alleviating hyperuricemia by degrading urate precursors. This study characterized *Enterococcus faecium* YC07 isolated from the traditional Chinese fermented food Jiangshui, which demonstrated efficient biodegradation of nucleosides (urate precursors), converting 2.0 g/L to nucleobases within 48 h. Whole genome sequencing revealed a 2.53 Mb draft genome (59 contigs, 38.21% GC content) containing 2387 protein-coding genes. Genomic and phenotypic analysis confirmed its probiotic potential, including high tolerance of simulated gastric fluid (98.89% survival) and intestinal fluid (44.51% survival), and strong adhesion capacity (24.16% auto-aggregation, 35.48% hydrophobicity), pathogen inhibition, and antioxidant activity. The identified antibiotic resistance genes and virulence factors were assessed alongside acute oral toxicology, cytotoxicity, antibiotics susceptibility, hemolysis, and enzymatic activity assays, confirming safety. Furthermore, successful pilot-scale fermentation in a 100 L fermenter demonstrated industrial feasibility. These findings established *E*. *faecium* YC07 as a safe and effective probiotic candidate for functional foods targeting hyperuricemia management.

## 1. Introduction

Jiangshui, a traditional fermented food in Northwest China, is made from a variety of vegetables such as rapeseed, celery, and radish leaves. In the Jiangshui fermentation process, the primary microorganisms involved are lactic acid bacteria (LAB), acetic acid bacteria, and yeast, with certain facultative anaerobic lactic acid bacteria being worth noting [1]. A previous questionnaire survey conducted among 180 residents in Lanzhou, China, revealed a negative correlation between the consumption of Jiangshui and the incidence of gout [2]. Building on these initial observations, researchers have taken a significant step forward by isolating bacterial strains from Jiangshui that exhibit the potential to reduce urate levels, including *Limosilactobacillus fermentum* JL-3 [2], *Limosilactobacillus fermentum* GR-3 [3], and *Lacticaseibacillus paracasei* JS-3 [4]. These findings highlight the significance of Jiangshui as a candidate for further research into reducing urate levels.

Urate, the end-product of purine metabolism, is the salt form of uric acid and is typically dissolved in the blood, and then filtered out by the kidneys and excreted in urine [5]. Under normal circumstances, the body maintains a balance between the production and excretion of urate [6]. However, hyperuricemia is caused by the overproduction or the underexcretion of uric acid, which is defined as a serum uric acid level above 6.8 mg/dl [7]. Hyperuricemia can lead to a series of complications, especially gout, a form of inflammatory arthritis that results from the deposition of urate crystals in joints and soft tissues [8].

Currently, the main approaches to improving hyperuricemia are divided into two categories: pharmacological treatment and dietary intervention [9]. However, the side effects of pharmacological treatment cannot be ignored, such as allergic reactions and potential damage to the liver and kidneys [10]. There are also indication limitations for drug therapy, especially for early-stage asymptomatic patients. Additionally, compliance issues may arise due to the complexity of long-term medication regimens and potential drug interactions [11]. On the other hand, dietary intervention requires sustained adherence to specific eating habits, which can be challenging and can lead to nutritional imbalances [12]. Its efficacy varies among individuals and is quite limited. Therefore, developing more effective and safe approaches for managing hyperuricemia is urgent and significant.

Probiotics, which are live microorganisms, could confer a health benefit on the host when consumed in adequate amounts (FAO/WHO [13]). Various microorganisms, including *Lactobacillus*, *Streptococcus*, *Bifidobacterium*, *Enterococcus*, *Bacillus*, and yeasts, have been reported that could ameliorate a variety of diseases, and an increasing number of studies are demonstrating the significant potential of probiotics in ameliorating hyperuricemia [14]. Probiotic strategies to ameliorate hyperuricemia aim to reduce urate biosynthesis and promote urate excretion. *Enterococcus faecium*, a species of LAB, is commonly found in natural environment, intestinal tract, and fermented foods [15]. Although *E*. *faecium* is currently approved for direct use in animal feed in China, *Enterococcus* species lack generally recognized as safe (GRAS) status for human applications [16]. Furthermore, as noted, this genus is currently not granted Qualified Presumption of Safety (QPS) status by the European Food Safety Authority (EFSA) [17]. Thus, further chronic toxicity studies and clinical trials are essential to establish its safety for human functional foods.

In this study, a bacterial strain with nucleosides-biodegrading capacity was isolated from Jiangshui and identified as *E*. *faecium* YC07. To provide a basis for developing functional food ameliorating hyperuricemia, we integrated whole genome sequencing, in silico safety profiling, and phenotypic validation to systematically evaluate YC07’s probiotic potential. We further demonstrated its industrial viability through high-density fermentation, bridging the gap between laboratory discovery and commercial application.

## 2. Materials and Methods

### 2.1. Isolation of the Bacterial Strain and Growth Conditions

*E*. *faecium* YC07 was isolated from traditional fermented Jiangshui obtained from a local market at Pingliang, China, using nucleosides as the sole carbon and energy source. The YC07 monoclonal colony activated in de Man, Rogosa and Sharpe (MRS) medium was then stored in 25% glycerol at −20 °C. The growth curve and pH curve were determined as follows. The activated strain was inoculated with 2% inoculum into MRS medium. The OD_600_ and pH values of the medium were determined at different time intervals. The growth or pH curve was plotted with the incubation time as the horizontal coordinate and OD_600_ or pH as the vertical coordinate. All of the experiments above were performed at 37 °C without shaking.

*Staphylococcus aureus* and *Escherichia coli* were provided by associate professor Yang Liu (Department of Biological Science and Engineering, University of Science and Technology Beijing, China), which were cultured in Luria–Bertani (LB) medium at 37 °C with a shaking rate of 200 rpm overnight.

### 2.2. Whole Genome Sequencing and Genome Annotation

The genomic DNA of strain YC07 (18-h-old) was extracted with a MagPure Bacterial DNA Kit (D6361-02, Magen, Shanghai, China), followed by assessment of its concentration, integrity, and purity. Whole genome sequencing was performed on the MGI DNBSEQ-T7 platform at Sangon Biotech (Shanghai, China). The resulting raw reads underwent quality control and adapter trimming using Trimmomatic (v0.36) to generate clean reads. The genome assembly utilized SPAdes (v3.15) coupled with Gapfiller (v1.11) for gap closure. Gene prediction and annotation were conducted with Prokka (v1.10) against the NCBI database. Functional annotation of the predicted protein-coding genes primarily relied on the COG (Cluster of Orthologous Groups of proteins), GO (Gene Ontology), and KEGG (Kyoto Encyclopedia of Genes and Genomes) databases. The VFDB (Virulence Factor Database) and CARD (Comprehensive Antibiotic Resistance Database) were queried to predict the virulence genes and antibiotic resistance genes.

### 2.3. Identification of YC07

Strain YC07 was phylogenetically characterized through combined 16S rRNA gene sequencing and average nucleotide identity (ANI) analysis [18]. For the ANI comparison, chromosomal sequences of nine reference strains were retrieved from GenBank (accessions detailed in Table S1). OrthoANI (https://www.ezbiocloud.net/tools/orthoani accessed on 15 October 2024) was employed to compute pairwise ANI values. Subsequently, a heatmap was constructed to visualize the resulting genetic similarities among the strains.

### 2.4. Biodegradation of Nucleosides by E. faecium YC07

To determine the nucleosides biodegrading capacity, 18-h-old *E*. *faecium* YC07 cultures were transferred to the new medium (0.50 g/L Na_2_HPO_4_, 0.05 g/L KH_2_PO_4_, 0.10 g/L MgSO_4_, 0.01 g/L CaCl_2_, 1.0 g/L inosine, 1.0 g/L guanosine, initial pH 7.2) and then biodegradation experiment was performed at 37 °C for 48 h. Subsequently, culture solution was taken at 24 and 48 h for determining the nucleosides concentration by HPLC (Shimadzu LC-20AT, Tokyo, Japan) [19]. The non-inoculated medium acted as the control.

### 2.5. Probiotic Characteristics

#### 2.5.1. Tolerance of Simulated Gastrointestinal Fluids

Simulated gastric fluid and intestinal fluid tolerance assays were determined according to a previous report, with modifications [20]. Briefly, 18-h-old YC07 cultures were centrifuged at 4 °C at 12,000 rpm for 15 min, washed twice using 0.01 M phosphate-buffered saline (PBS, pH 7.2), and resuspended in simulated gastric fluid (2.0 g/L NaCl, 3.0 g/L Pepsin, pH adjusted to 2.5 using 0.50 M HCl) and simulated intestinal fluid (1.0 g/L Trypsin, 3.0 g/L bile salt, pH adjusted to 8.0 using 0.50 M NaOH). The cell suspension was incubated at 37 °C for 3 h and 5 h, respectively. The control experiment was that YC07 was then harvested and resuspended in stroke-physiological saline solution. Survival rates were calculated as (%) = (Final (log CFU/mL)/Initial (log CFU/mL)) × 100.

#### 2.5.2. Antimicrobial Activity

According to a previous report, with modifications [21], the Oxford cup method was used to measure the antimicrobial activity. Cell-free supernatant (CFS) was generated by centrifuging (10,000 rpm, 4 °C, 15 min) the 48 h fermentation broth of *E*. *faecium* YC07, followed by filtration through a 0.22 μm membrane. Activated cultures of *S*. *aureus* and *E*. *coli* were mixed into sterilized LB agar cooled to approximately 40 °C. After solidification, sterilized Oxford cups were aseptically placed onto the agar surface. Each cup received 200 μL of CFS, which was allowed to diffuse at 4 °C for 6 h. The plates were then incubated at 37 °C for 24 h. Following the formation of inhibition zones around the wells, the diameters of the clear zones were measured using a vernier caliper.

#### 2.5.3. Auto-Aggregation and Cell Surface Hydrophobicity

Auto-aggregation and cell surface hydrophobicity were performed as described by Seddik et al. [22]. Briefly, *E*. *faecium* YC07 was grown for 18 h at 37 °C in MRS medium. After centrifugation (12,000 rpm, 15 min) and resuspension, the cell suspensions (10^8^ CFU/mL) were mixed by vortexing and incubated 37 °C for 4 h. At the same time, xylene was added to the cell suspension (*v*:*v* = 1:3) and mixed by vortexing for 2 min. And then, the YC07 suspension was incubated at room temperature until phase separation. The absorbance of the liquid phase of the suspension was detected by a spectrophotometer (INNESA, Shanghai, China) at 600 nm. The auto-aggregation or cell surface hydrophobicity percentages were calculated as (%) = 1 − (A_t_/A_0_) × 100, where A_t_ represented the final absorbance or aqueous phase after phase separation, and A_0_ showed the initial absorbance.

#### 2.5.4. Evaluation of the Antioxidant Activity

*E*. *faecium* YC07 was grown and centrifuged as described in the above section to isolate the cells and fermentation supernatant. The cells were washed and suspended in sterile saline to adjust the concentration 10^9^ CFU/mL, which was considered as the bacterial suspension. The TAC of the cell suspension and fermentation supernatant were determined according to the method guidance of the FRAP method kit (Acmec biochemical, Shanghai, China).

### 2.6. Safety Assessment

#### 2.6.1. Hemolytic Activity

*E*. *faecium* YC07 and *S*. *aureus* were plated on Columbia blood agar plates. After incubation at 37 °C for 48 h, signs of β-hemolysis (complete hemolysis), α-hemolysis (incomplete hemolysis), or γ-hemolysis (non-hemolysis) were observed [23].

#### 2.6.2. Antibiotic Resistance

The susceptibility of *E*. *faecium* YC07 to eight common antibiotics, namely, erythromycin (15 μg), kanamycin (30 μg), gentamicin (10 μg), vancomycin (30 μg), chloramphenicol (30 μg), tetracycline (30 μg), ciprofloxacin (5 μg), and ampicillin (10 μg) (BKMAM, Changsha, China), was determined using Kirby–Bauer method recommended by the Clinical and Laboratory Standards Institute (CLSI) M100 [24].

#### 2.6.3. Biogenic Amine Production Assay

The biogenic amine production assay of the *E*. *faecium* YC07 was performed using a micro-biochemical identification tube for bacteria (Hopebio, Qingdao, China). Briefly, YC07 cell suspension (100 μL) was inoculated into the amino acid decarboxylase control broth, lysine decarboxylase broth, ornithine decarboxylase broth, and L-arginine decarboxylase broth. After that, sterilized liquid paraffin (300 μL) was added into each tube and then incubated at 37 °C for 24 h. The color change in the broth was observed. The appearance of purple in the broth indicated that the corresponding biogenic amine was produced.

#### 2.6.4. Gelatinase Activity

The Gelatinase activity assay was performed using a micro-biochemical identification tube for bacteria (Hopebio, Qingdao, China). Briefly, the cell suspension was obtained using the method consistent with Section 2.5.3, and then 100 μL of the cell suspension was inoculated into gelatin biochemical tubes and incubated at 37 °C for 48 h. After that, we let it stand at 4 °C for 30 min and observed its state. The content of the remaining liquid was evaluated as positive.

#### 2.6.5. Acute Oral Toxicology Test and Cytotoxicity Assay

To prepare *E*. *faecium* YC07 lyophilized powders, the pilot-scale fermentation was conducted in a 100 L fermenter (Bailun bio, Shanghai, China) using MRS broth as the basal medium. To ensure process reproducibility and optimal growth conditions, the pH was monitored continuously throughout the fermentation. When the pH decreased below 6.0, fed-batch additions of sterile glucose solution (10 g/L final concentration) and aqueous ammonia (as both nitrogen source and pH regulator) were administered. This strategy maintained the pH above 6.0 at all times. Samples were collected aseptically every 2 h for offline analysis, including OD_600_ measurements (to monitor biomass growth) and microscopic examination (to assess cell morphology and purity). When the OD_600_ value exceeded 6, the cells were harvested by centrifuging (at 12,000 rpm) at room temperature and then freeze-dried and crushed.

Twelve specific pathogen-free (SPF) ICR mice (50% male, 21–22 g) were obtained from Wuhan Halic Bio-technology Co., Ltd. (Wuhan, China). Freeze-dried *E*. *faecium* YC07 powder was resuscitated and cultured in MRS broth to achieve a concentration of 10^7^ CFU/mL. Using the limit test method, a single dose of 20 mL/kg body weight (BW) was administered. Following a 7-day acclimatization period, the mice were fasted for 4 h with water ad libitum. The experimental group then received a single oral gavage of the bacterial suspension at 0.2 mL/10 g BW. The mice were housed under controlled temperature and humidity conditions with access to a standard laboratory diet. The 14-day observation period concluded with the immediate assessment of toxicity classification after the final administration [25].

The cytotoxicity of *E*. *faecium* YC07 was tested in a human colorectal adenocarcinoma epithelial cell line (IPEC-J2). IPEC-J2 cells were grown in Minimum Essential Medium (MEM) supplemented with 20% (*v*/*v*) foetal bovine serum at 37 °C and 5% CO_2_ to the logarithmic growth phase. Cells were detached using Trypsin (0.25%) and we then made the cell suspension. IPEC-J2 cells were inoculated into 96-well plates (5000/well) and cultured in MEM medium for 24 h. The YC07 cultures were centrifuged (10,000 rpm, 10 min) and resuspended in MEM medium. Following this, the MEM medium in the 96-well plate was replaced with *E*. *faecium* YC07 cell suspension (10^7^ CFU/mL), and the cells were incubated for 24 h. Subsequently, 10 μL of CCK-8 reagent was added to each well containing 100 μL of the culture medium, followed by a further 4 h incubation. Blank control wells received an equivalent volume of fresh MEM medium. Absorbance at 450 nm was measured, and cell viability (%) was calculated using the formula 100 × (A/Ab), where A represents the absorbance of the wells containing cells and bacteria, and Ab represents the absorbance of the blank control wells [18].

### 2.7. Statistical Analysis

All the analyses were carried out in three parallel experiments, and the results are expressed as means ± SD. The statistics were analyzed using Excel Office and Origin 2021.

## 3. Results and Discussion

### 3.1. Isolation of Nucleoside Biodegrading Strain YC07 and Product Identification

**Figure 1:**
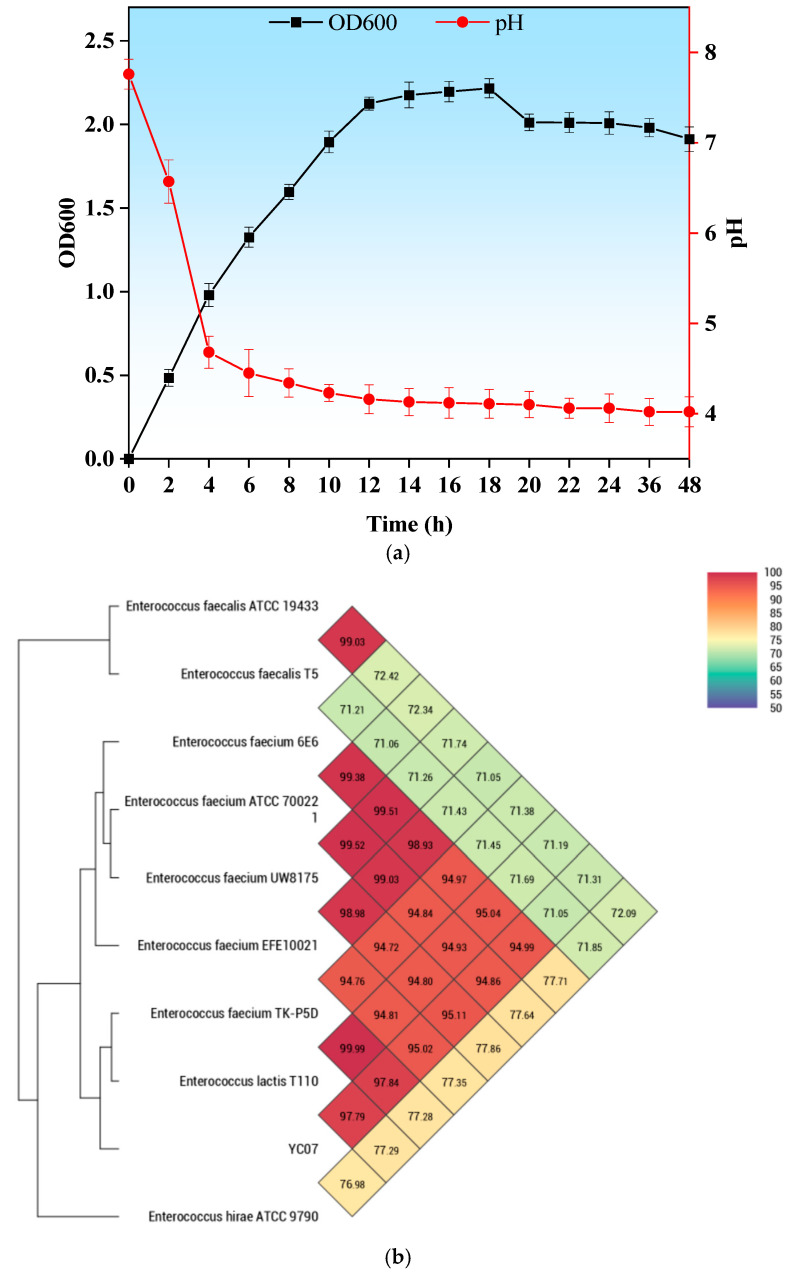
(**a**) Isolation, characterization, and identification of *E*. *faecium* YC07. The growth curve and pH curve of *E*. *faecium* YC07; (**b**) Heatmap generated indicating Orthologous Average Nucleotide Identity values calculated between *E*. *faecium* YC07 and other closely related *Enterococcus* genera.

Strain YC07 capable of biodegrading nucleosides was isolated from Jiangshui; its colony is convex and circular, with a diameter of approximately 1.0–1.5 mm, white and opaque (Figure S1a). The YC07 cell morphology were spherical, occurring singly, in pairs, or in short chains, and sometimes extended into long chains (Figure S1b). The OD_600_ and pH values represented the growth rate and acid production of YC07 (Figure 1a), showing an OD_600_ value above 1.6 and pH value about 4.0 within a 48 h incubation period. In addition, 16S rRNA gene sequencing identified YC07 as a member of the *Enterococcus* genus, consistent with its observed coccoid morphology under microscopy. Furthermore, ANI analysis revealed 97.84% similarity between the YC07 genome and the *Enterococcus faecium* type strain genome (Figure 1b).

*E*. *faecium* and *E*. *faecalis* are the two predominant species of *Enterococcus* in different traditional fermented foods, such as fermented vegetable foods [26], dairy products [27], and fermented soybean products [23]. Generally, *E*. *faecium* showed fewer virulence factors than *E*. *faecalis*, which appears to pose a lower risk for use in foods [28]. At present, some strains such as *E*. *faecium* M74 and *E*. *faecium* SF-68 are included as food supplements in several probiotic preparations that have been proved to be effective and safe [16]. The probiotic potential and safety concerns surrounding enterococci remain subjects of ongoing scientific debate. Therefore, it is essential to evaluate the safety and probiotic characteristics of *E*. *faecium* YC07.

**Figure 2:**
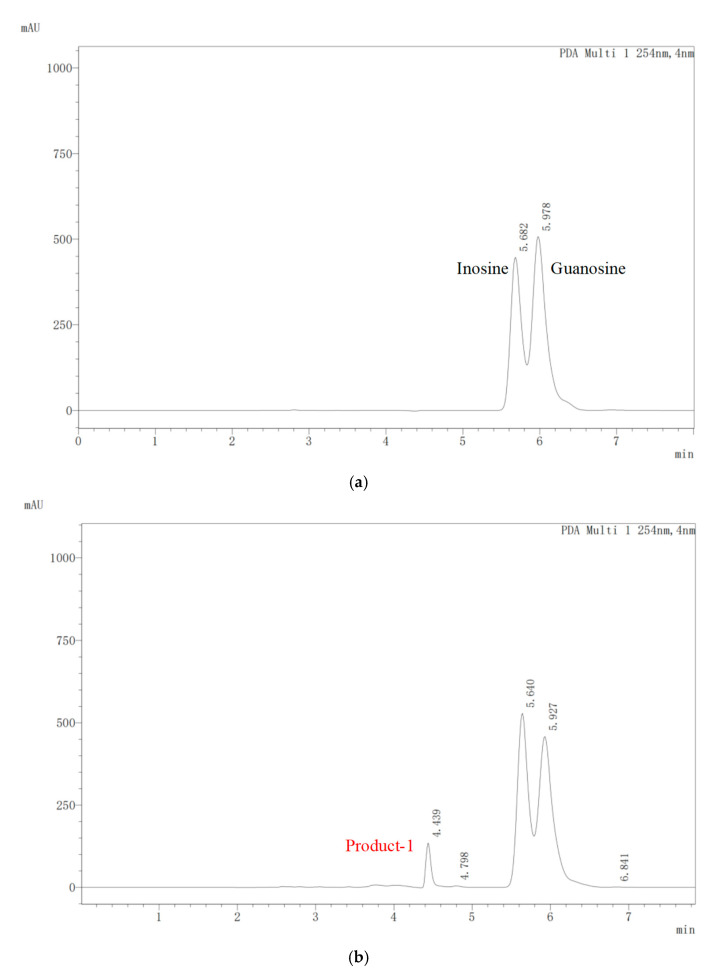

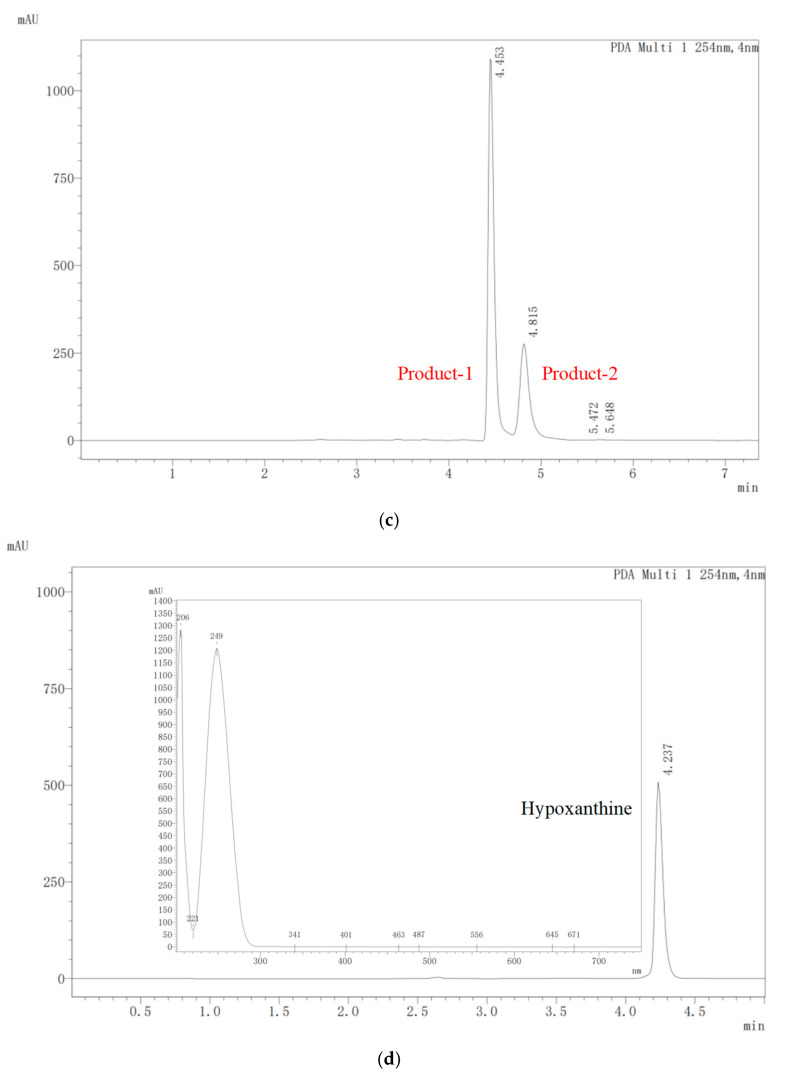

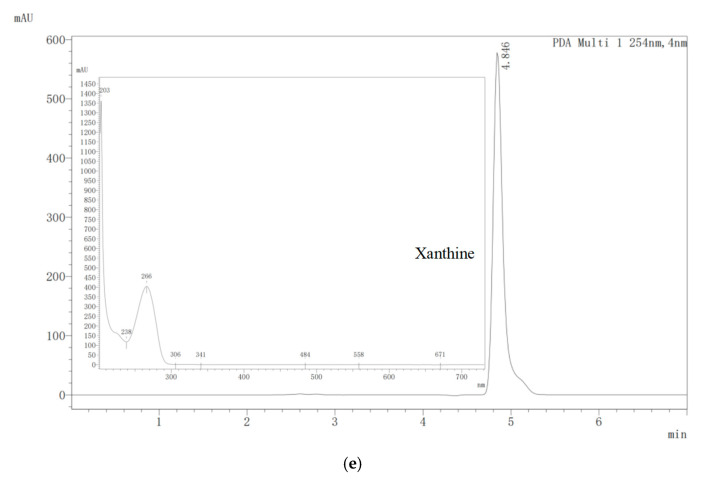
The nucleoside (both inosine and guanosine) biodegradation products of *E*. *faecium* YC07 detected by HPLC. Nucleoside biodegradation and metabolite production at 0 h (**a**), 24 h (**b**), and 48 h (**c**) by YC07. Detection peak of hypoxanthine standard (**d**) and xanthine standard (**e**).

To analyze the urate-lowering potential of *E*. *faecium* YC07, it was added to the medium using both inosine and guanosine (2 g/L in total) as the sole carbon and nitrogen source for cultivation. The HPLC results showed that YC07 could biodegrade nucleosides and completely convert them within 48 h (Figure 2a–c). The HPLC result for the 24 h sampling showed an absorption peak of biodegradation product at 4.439 (Figure 2b). During the subsequent biodegradation process, this product progressively increased, and a new biodegradation product (absorption peak at 4.815) emerged (Figure 2c). These two products were consistent with the standard of hypoxanthine peak (Figure 2d) and xanthine peak (Figure 2c). These results suggested that YC07 biodegraded inosine and guanosine into smaller molecules such as hypoxanthine and xanthine. The prevalence of hyperuricemia is gradually increasing and the patients becoming younger in the world, which is influenced by variety factors. The present studies focused on the probiotics isolated from fermented food to improve hyperuricemia. Probiotics reduce the intestinal epithelial cells’ absorption of purine compounds through degrading nucleosides into nucleobases, which consequently decreases the urate production. Based on the HPLC results, we have preliminarily determined that *E*. *faecium* YC07 is capable of biodegrading nucleosides into hypoxanthine and xanthine. Emerging research indicates that certain probiotic strains catabolize nucleosides, reducing levels of metabolites like ribose and purine bases, which decrease in metabolic intermediates and subsequently attenuate urate synthesis [29]. As primary probiotics, *Lactobacillus* spp. attenuate serum uric acid levels via nucleoside biodegradation, such as *Lacticaseibacillus rhamnosus* Fmb14 [30], *L*. *acidophilus* F02 [31], *L*. *fermentum* 9-4 [32], and *Levilactobacillus brevis* PDD-5 [33]. All of these strains are isolated from different fermented foods, including yogurt, fermented rice-flour noodles and pickles. At present, probiotics are recognized for their potential to alleviate hyperuricemia through multiple pathways. In this study, we have preliminarily discovered the ability of *E*. *faecium* YC07 to biodegrade urate precursors in vitro. However, a series of subsequent experiments are necessary to explore its potential for improving hyperuricemia in vivo, such as mouse model studies.

### 3.2. Genome Feature of E. faecium YC07

Whole genome sequencing and function annotation were performed on the *E*. *faecium* YC07. The total number of bases after quality control was 1.31 G. The integrity of the initial genome sequence assemblies was evaluated, and the outcomes indicated a high-quality assembly, which is suitable for subsequent detailed analyses (Figure S2). The whole genome sequence of YC07 was 2.5 Mb, with an average GC content of 38.19%. The reads were assembled into 59 contigs with an N50 of 98,733 bp. The number of the predicted protein-coding genes (CDSs) was 2387, and genome analysis revealed that 54 tRNA genes, 6 rRNA genes, and 1 ncRNA gene were predicted. All the detailed parameters are shown in Table S2; *E*. *faecium* YC07 had similar genomic GC content and genome size compared with other reported *Enterococcus* strains [34].

**Figure 3:**
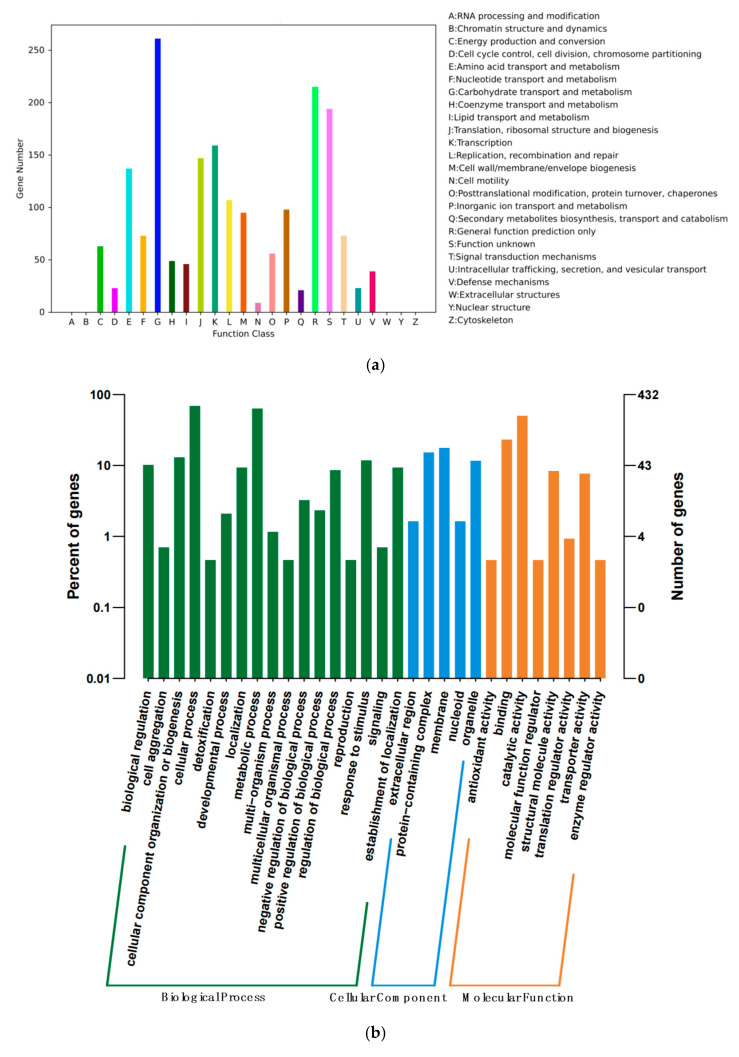

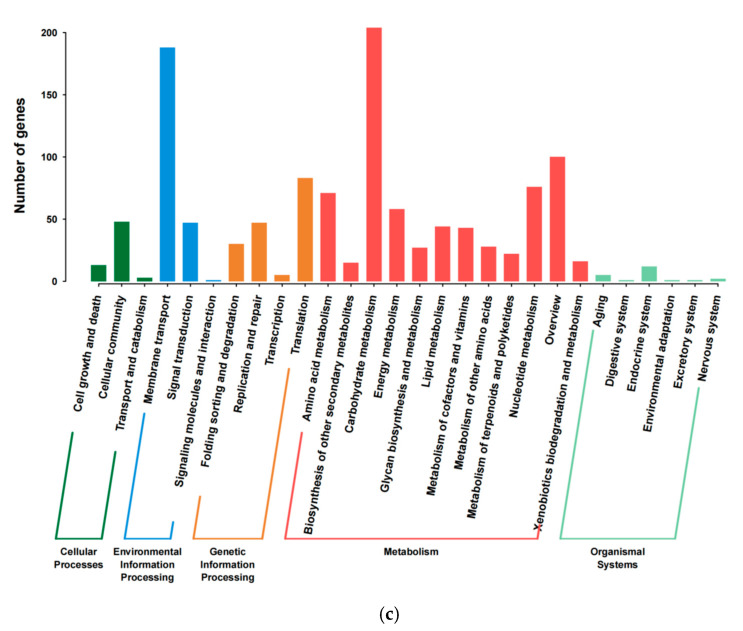
Statistical legend of gene annotation classification of *E*. *faecium* YC07. (**a**) COG function classification; (**b**) GO function classification; (**c**) Histogram of KEGG.

Of the 2387 CDSs, 1749 genes could be categorized into 21 different categories of COG (Figure 3a), which were focused on the function of nucleotide transport and the metabolism matched 122 genes. Notably, 779 genes of YC07 were annotated in the KEGG database (Figure 3b), which was involved in five categories. In addition, 432 CDSs of YC07 were annotated in GO (Figure 3c).

Following the above biodegradation product discovery, we further analyzed the whole genome sequencing information to elucidate the biodegradation pathways at the genetic level. The genes *rihB*, *punA*, and *deoD* were detected in the YC07 genome, encoding for nucleoside hydrolase and purine nucleoside phosphorylase, which are responsible for biodegrading nucleosides [35]. In addition, whole genome sequencing information analysis has shown that multiple genes play a key role in survival in harsh conditions, antibacterial substance, and intestinal colonization (Table 1).

### 3.3. Simulated Gastrointestinal Fluid Tolerance Assays

Probiotics confer a healthy benefit on the host when administered in adequate amounts. Therefore, a probiotic candidate must survive and thrive in the harsh conditions of the gastrointestinal tract, including resisting gastric acid, bile salt, pepsin, and trypsin [36]. The tolerance of *E*. *faecium* YC07 to simulated gastrointestinal fluids was tested. When incubated in simulated gastric fluid for 3 h and simulated intestinal fluid for 5 h, the YC07 demonstrated ideal gastrointestinal fluid tolerance, exhibiting a survival rate of 98.89% and 44.51%, respectively. Similarly, previous research reported that the survival rates of *E*. *faecium* strains in simulated gastric fluid (0.3% pepsin and 0.5% saline, pH 3) were between 96.31 and 99.13% [37]. On the other hand, *E*. *faecium* BH04, BH12, BH84, and BH99 could survive in simulated gastric fluid at rates of 79.25, 82.05, 76.80, and 82.05%, respectively [20]. It is known that *E*. spp. are more resistant to low pH than *Lactobacillus* spp. and *Lactococcus* spp. [22]. Many studies have shown that survival of *Enterococcus* strains decline as bile salt concentrations increase. In a previous study, during 5 h incubation in simulated intestinal fluid (3% bile salts, 1% pancreatin, pH 8), the count of *E*. *lactis* JDM1 viable bacteria remained stable at 10^3^–10^4^ CFU/mL [18]. In a separate investigation, both cheese-derived *E*. *faecium* ES4 and yoghurt-isolated *E*. *faecium* ES27 demonstrated substantial bile salt tolerance, maintaining viability rates of 70.1% and 68.3%, respectively, following 4 h exposure to 0.3% bile salt-containing medium at 37 °C [38]. Compared to the previous studies, *E*. *faecium* YC07 showed favorable tolerance of simulated gastrointestinal fluids.

### 3.4. Inhibition Ability of YC07 to Pathogens

The bacteriostatic ability of *E*. *faecium* YC07 against two common pathogenic bacteria strains was tested. The fermentation supernatant of the YC07 showed inhibitory activity against *S*. *aureus* and *E*. *coli* (Figure S3), and it showed better inhibition of *E*. *coli*. A probiotic strain may inhibit the growth of pathogens through producing antimicrobial substances like lactic acid, bacteriocins, and hydrogen peroxide [39]. However, the inhibitory effects observed in vitro may not automatically equate to in vivo effectiveness. The human gastrointestinal tract is an intricate environment, and the efficacy of probiotics is subject to a multitude of factors [40]. Additional research, particularly animal studies, is necessary to validate YC07’s ability to curb pathogen proliferation or infection within a living system.

### 3.5. Auto-Aggregation and Cell Surface Hydrophobicity

The findings indicate that the auto-aggregation capability of the *E*. *faecium* YC07 was 13.76 ± 1.73% after 2 h and 24.16 ± 0.85% after 4 h, showing an increase over time. On the other hand, we determined that the hydrophobicity of *E*. *faecium* YC07 to xylene was 35.48% ± 1.45%. As a probiotic candidate, *E*. *faecium* YC07 must be able to colonize the host’s intestinal mucosa to be effective. Auto-aggregation refers to the tendency of the same bacterial strains to cluster together, while hydrophobicity refers to the bacteria’s capacity to adhere to surfaces [22]. In a previous study, the auto-aggregation capacity of *E*. *faecium* strains were 11.95–21.05%, and hydrophobicity could not be detected [20]. As a result, the *E*. *faecium* YC07 in the present study could be thought have good adhesion.

### 3.6. Antioxidant Activity

The total antioxidant capacity (TAC) of the fermentation supernatant and bacterial suspension were 1.49 ± 0.25 μmol/mL and 0.21 ± 0.06 μmol/mL, respectively. A higher antioxidant ability was identified in the fermentation supernatant, which might be due to the metabolites secreted outside the cells. Antioxidant capacity in the context of probiotic characteristics evaluation refers to the collective ability of a probiotic strain to counteract oxidative stress and neutralize free radicals, which are unstable molecules that can cause damage to cells and tissues [41]. Research has established that specific probiotic strains are capable of colonizing the intestinal tract and functioning as antioxidants, thereby preserving the intestinal redox balance [42]. These beneficial microbes produce metabolites that can mitigate oxidative stress, which is a contributing factor to aging and numerous chronic diseases [43]. It is apparent that YC07 exhibits antioxidant capabilities, likely attributed to the presence and expression of genes conferring resistance to oxidative stress within its genome, such as NADH peroxidase, NADH oxidase, NADH dehydrogenase, superoxide dismutase, hydroperoxide reductase, thioredoxin, thioredoxin reductase, and thiol peroxidase.

### 3.7. Safety Properties

#### 3.7.1. Safety-Related Gene Profile

In the present study, putative antibiotic resistance genes were detected in the *E*. *faecium* YC07 genome and the results are given in Table 2. *E*. *faecium* YC07 carries the conserved gene *aac(6′)-Ii*, which encodes an aminoglycoside acetyltransferase. In addition, *efmA* was found in the YC07, which is the MFS transporter permease that confers resistance to fluoroquinolone and macrolide antibiotics. However, as detailed in Section 3.7.2, subsequent in vitro susceptibility testing demonstrated that the strain YC07 exhibits resistance only to erythromycin (a macrolide) among the eight common antibiotics tested. This highlights the necessity of experimental validation for genomic predictions of antibiotic resistance.

Eight predicted virulence factors were found in the genome of the *E*. *faecium* YC07 (Table 3), which are not aggressive. The enterococcal virulence factors (*cylA*, *Asa*, *esp*, *efaA*, *hyl*, and *gelE*), which were also found in previous studies, play an important role in the pathogenicity of the *Enterococcus* genus, including enhancing the bacteria’s ability to adhere, invade, and evade host immune responses. However, none of these was discovered in the *E*. *faecium* YC07. Virulence factors are molecules produced by pathogens that enhance their ability to cause disease [20], which include adhesins that allow the pathogen to attach to host cells, toxins that damage host tissues, and proteins that help the pathogen evade the host’s immune system [44].

#### 3.7.2. Antimicrobial Susceptibility Test

The *E*. *faecium* YC07 only showed resistance to erythromycin, and exhibited sensitivity to kanamycin, gentamicin, vancomycin, chloramphenicol, tetracycline, ciprofloxacin, and ampicillin. To meet probiotic safety criteria, strains must exhibit negligible antibiotic resistance. The strain YC07 demonstrates strong potential in this regard, showing susceptibility to most antibiotics evaluated in this study. In addition, the in vitro experiment results also corroborate the findings from the genomic analysis. Antibiotic resistance is a critical global health challenge, whereby bacteria and other microorganisms evolve to withstand the effects of antibiotics, rendering these life-saving drugs ineffective [45]. The FAO/WHO reported that *Enterococcus* spp. are increasingly vancomycin resistant [16], but no related genes were found in the YC07 genome. From the whole genome sequencing results, it appears that YC07 harbors a limited number of antibiotic resistance genes, which theoretically suggests a certain level of safety. However, it is essential to note that theoretical predictions must be corroborated through further in vitro experiments to validate the strain’s safety profile. The result is important to this strain’s potential use in the food industry.

### 3.8. In Vitro Safety Assessment of E. faecium YC07

Hemolysis activity in a safety assessment refers to the ability of certain microorganisms to produce hemolysins, which are toxins that can destroy the membrane of red blood cells, leading to the release of cellular contents [37]. This capability serves as one of the indicators for evaluating the pathogenicity and potential hazards of microorganisms. After incubation on a Columbia blood agar plate for 48 h, the *E*. *faecium* YC07 exhibited no hemolysis ability (Figure S4a) compared with *S*. *aureus*, which produced strong β-hemolysis (Figure S4b).

The biogenic amine production by *E. faecium* YC07 was assessed in decarboxylase broths supplemented with three precursor amino acids. The strain tested negative for lysine and ornithine decarboxylation but positive for arginine decarboxylation (purple color development, Figure S3c). This indicates that YC07 expresses functional arginine decarboxylase, converting arginine to agmatine. Biogenic amines are a collection of low-molecular-weight organic compounds that can cause toxicity when consumed in large amounts [46]. The primary source of these amines in food is their production through microbial decarboxylation processes. However, agmatine is a ubiquitous biogenic amine that plays an important role in cellular processes, and it does not have a clear health-harming effect, but when combined with nitrogen, it can form nitrites, enhancing the toxicity of histamine and tyramine [47]. Biologically significant amines, such as histamine, putrescine, cadaverine, tyramine, tryptamine, spermine, and spermidine, are prevalent in food. Nevertheless, the genomic analysis of the *E*. *faecium* YC07 revealed the absence of the corresponding decarboxylase genes associated with these amines.

In addition, the results showed that the YC07 did not have gelatinase activity. Gelatinase activity is another significant indicator in the safety assessment, particularly as it relates to pathogenicity and spoilage potential [20]. Understanding the hemolytic potential and gelatinase activity is crucial in evaluating microorganisms’ potential risks to food safety. These tests are fundamental in determining the safety and functionality aspects of *E*. *faecium* YC07, which are desirable for its use in food products. Therefore, it is thought that the *E*. *faecium* YC07 isolates used in this study will not cause any health problems.

### 3.9. Acute Oral Toxicology and Cytotoxicity Tests

**Figure 4:**
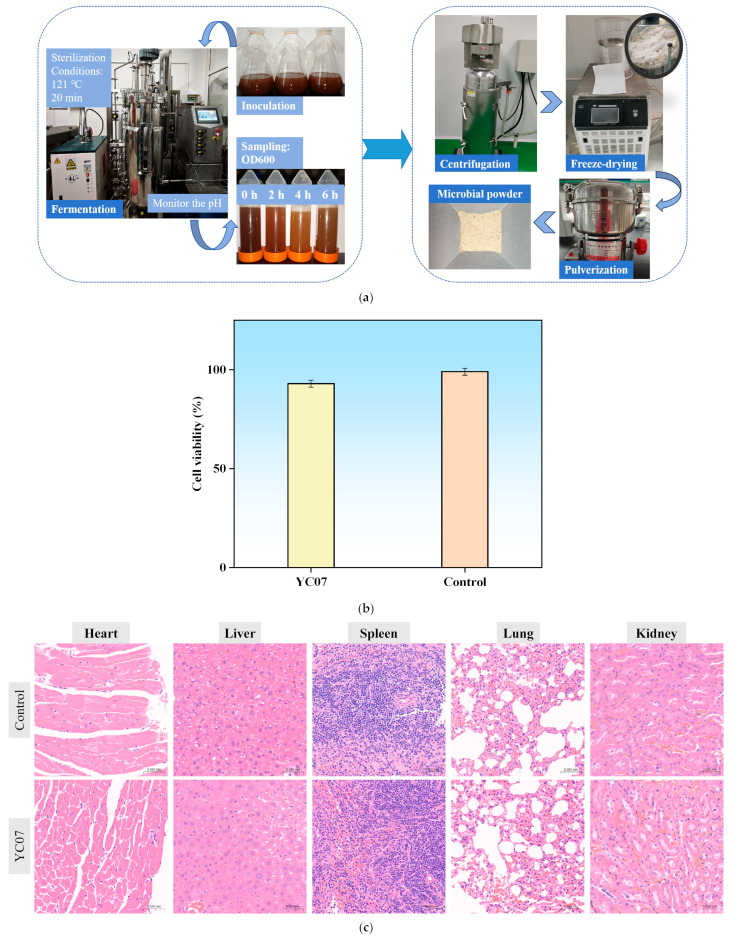
(**a**) The pilot-scale fermentation process; (**b**) Cytotoxicity assay of *E*. *faecium* YC07; (**c**) Safety assessment of *E*. *faecium* YC07, histological section of heart, liver, spleen, lung, and kidney.

A pilot-scale fermentation was conducted (Figure 4a), which ultimately obtained approximately 80 g of high-density (3.18 × 10^9^ CFU/g) bacterial powder. This experiment primarily aimed to provide YC07 samples for subsequent acute toxicity and cytotoxicity tests. Meanwhile, we could make clear and define the potential of this strain for future applications in the food industry through this trial.

The cytotoxicity of the *E*. *faecium* YC07 toward human colorectal adenocarcinoma epithelial (IPEC-J2) cells was assessed. Following 24 h incubation with bacterial suspension in MEM medium, no significant decrease in cell viability was observed versus the controls (Figure 4b). Consistent with the >80% viability threshold for non-cytotoxicity, these results indicate that YC07 lacks significant cytotoxic effects on IPEC-J2 cells.

The mice were fed once with 20 mL/kg BW of *E*. *faecium* YC07 cultures. The weights of the mice had remained stable to some extent during the 14 days of observation and no poisonings or deaths were found (Table 4). After the experiment, the mice were dissected and no abnormalities were found (Figure 4c). According to the GB 15193.3-2014 “National Food Safety Standards-Acute Oral Toxicity Test”, the acute oral LD50 of YC07 (10^7^ CFU/mL) in the ICR mice was more than 20 mL/kg BW, which indicated that *E*. *faecium* YC07 can be classified as a non-toxic grade.

For a probiotic candidate, acute oral toxicity and cytotoxicity tests are intended for the development of functional food products in the future. The results indicated that the *E*. *faecium* YC07 had no toxic effect. Pilot-scale fermentation tests lay the foundation for the subsequent factory level fermentation. However, its urate-lowering potential has so far only been demonstrated through the biodegradation of urate precursors in vitro. Future research will focus on establishing a hyperuricemia mouse model and feeding the mice with YC07 powder to observe whether the symptoms can be improved. Based on the current research status, we will also investigate whether YC07 can inhibit xanthine oxidase activity and regulate the expression of urate transporters, as well as whether it can ameliorate kidney damage caused by hyperuricemia [14]. According to T/CIFST 009-2022 “General standard of probiotics for food use”, these studies will provide valuable insights into the strain’s efficacy and safety, which are paramount for determining its suitability as a probiotic intervention for hyperuricemia.

## 4. Conclusions

In this study, we comprehensively characterized *Enterococcus faecium* YC07, a Jiangshui-derived probiotic candidate, through integrated genomic and experimental approaches. Genomic analysis identified key probiotic-associated genes and nucleoside hydrolase genes that correlated with its observed phenotypic traits: robust nucleoside biodegradation (efficient conversion of 2.0 g/L nucleosides within 48 h), gastrointestinal tolerance, pathogen inhibition, and antioxidant capacity. Crucially, the safety of this strain was preliminarily verified through in vitro and in vivo tests. The pilot-scale fermentation achieved industrial-relevant biomass (10^9^ CFU/g), demonstrating scalable production feasibility. This multi-omics validation, spanning genotypic prediction, phenotypic corroboration, and industrial compatibility, establishes *E*. *faecium* YC07 as a promising probiotic candidate, providing profound insight into developing functional foods for ameliorating hyperuricemia. Future studies will establish a hyperuricemic mouse model to further investigate the in vivo potential of YC07 in ameliorating hyperuricemia.

## Figures and Tables

**Table 1:** Probiotic characteristic-related genes in *E*. *faecium* YC07 genome.

Gene ID	Location	Gene Name	Product
Antimicrobial activity			
gene0082	Contig1	*msrA*	Peptide methionine sulfoxide reductase MsrA
gene0413	Contig3	-	hypothetical protein
gene1641	Contig13	*btuD*	Vitamin B12 import ATP-binding protein BtuD
gene2177	Contig23	-	hypothetical protein
gene2464	Contig43	-	hypothetical protein
gene2465	Contig43	*lagD*	Lactococcin-G-processing and transport ATP-binding protein LagD
Acid resistance			
gene1045	Contig6	*atpC*	ATP synthase epsilon chain
gene1046	Contig6	*atpD*	ATP synthase subunit beta
gene1047	Contig6	*atpG*	ATP synthase gamma chain
gene1048	Contig6	*atpA*	ATP synthase subunit alpha
gene1049	Contig6	*atpH*	ATP synthase subunit delta
gene1050	Contig6	*atpF*	ATP synthase subunit b
gene1051	Contig6	*atpE-1*	ATP synthase subunit c
gene1052	Contig6	*atpB*	ATP synthase subunit a
gene1648	Contig13	*atpE-2*	V-type proton ATPase subunit E
gene0004	Contig1	*tyrS1*	Tyrosine--tRNA ligase 1
gene0001	Contig1	*nhaC-1*	Na(+)/H(+) antiporter NhaC
gene1755	Contig15	*nhaC-2*	Na(+)/H(+) antiporter NhaC
gene1075	Contig7	*asd*	Aspartate-semialdehyde dehydrogenase
gene0065	Contig1	*panP*	Aspartate 1-decarboxylase
gene0261	Contig2	*panT-1*	Pantothenate transporter PanT
gene1759	Contig15	*panE*	2-dehydropantoate 2-reductase
gene1799	Contig15	*panT-2*	Pantothenic acid transporter PanT
gene1146	Contig7	*arcD1-1*	Arginine/ornithine antiporter ArcD1
gene1477	Contig11	*arcC1-1*	Carbamate kinase 1
gene1478	Contig11	*arcB*	Ornithine carbamoyltransferase, catabolic
gene1479	Contig11	*arcA*	Arginine deiminase
gene1767	Contig15	*arcD1-2*	Arginine/ornithine antiporter ArcD1
gene2459	Contig41	*arcC1-2*	Carbamate kinase 1
gene0857	Contig5	*argR-1*	Arginine repressor
gene1101	Contig7	*argR-2*	Arginine repressor
gene1472	Contig11	*argR-3*	Arginine repressor
gene1475	Contig11	*argS*	Arginine-tRNA ligase
Bile salt tolerance			
gene1519	Contig11	*cbh*	Choloylglycine hydrolase
gene0588	Contig3	*ecsA*	ABC-type transporter ATP-binding protein EcsA
gene1078	Contig7	*patA*	Putative N-acetyl-LL-diaminopimelate aminotransferase
gene1438	Contig10	*patB*	Cystathionine beta-lyase PatB
Oxidative stress			
gene1079	Contig7	*npr*	NADH peroxidase
gene0033	Contig1	*nox-1*	NADH oxidase
gene0234	Contig2	*nox-2*	NADH oxidase
gene1628	Contig13	*nox-3*	NADH oxidase
gene0777	Contig5	*sodA*	Superoxide dismutase [Mn]
gene0988	Contig6	*trxB*	Thioredoxin reductase
gene1639	Contig13	*trxA*	Thioredoxin
gene2439	Contig38	*tpx*	Thiol peroxidase
gene0367	Contig2	*ahpC*	Alkyl hydroperoxide reductase C
gene0368	Contig2	*ahpF*	NADH dehydrogenase
Adhesion and aggregation			
gene0290	Contig2	*yloA*	putative protein YloA
gene1275	Contig8	*tuf*	Elongation factor Tu
gene1324	Contig9	*pdhA*	Pyruvate dehydrogenase E1 component subunit alpha
gene1325	Contig9	*pdhB*	Pyruvate dehydrogenase E1 component subunit beta
gene1327	Contig9	*pdhD*	Dihydrolipoyl dehydrogenase
gene1674	Contig13	*eno*	Enolase
gene0219	Contig2	*gap-1*	Glyceraldehyde-3-phosphate dehydrogenase
gene1671	Contig13	*gap-2*	Glyceraldehyde-3-phosphate dehydrogenase
gene1673	Contig13	*tpiA*	Triosephosphate isomerase
Ionic and heavy metal stress resistance			
gene0779	Contig5	*czcD*	Cadmium, cobalt and zinc/H(+)-K(+) antiporter
gene0984	Contig6	*zur*	Zinc-specific metallo-regulatory protein
gene1571	Contig12	*znuA*	High-affinity zinc uptake system binding-protein ZnuA
gene1572	Contig12	*znuC*	High-affinity zinc uptake system ATP-binding protein ZnuC
gene1573	Contig12	*znuB*	High-affinity zinc uptake system membrane protein ZnuB
gene2300	Contig28	*fetB*	putative iron export permease protein FetB
gene1167	Contig7	*iscU*	Iron-sulfur cluster assembly scaffold protein IscU
Temperature stress			
gene2279	Contig27	*hslO*	33 kDa chaperonin
gene1820	Contig16	*ctsR*	Transcriptional regulator CtsR
gene0238	Contig2	*dnaD*	DNA replication protein DnaD
gene0606	Contig4	*dnaK*	Chaperone protein DnaK
gene0607	Contig4	*dnaJ-1*	Chaperone protein DnaJ
gene0698	Contig4	*dnaB*	Replication initiation and membrane attachment protein
gene0699	Contig4	*dnaI*	Primosomal protein DnaI
gene1067	Contig7	*dnaJ-2*	Chaperone protein DnaJ
gene2032	Contig19	*dnaE*	DNA polymerase III subunit alpha
gene2059	Contig20	*dnaA*	Chromosomal replication initiator protein DnaA
gene2060	Contig20	*dnaN*	Beta sliding clamp
gene2070	Contig20	*dnaC*	Replicative DNA helicase
gene0605	Contig4	*grpE*	Protein GrpE
gene0604	Contig4	*hrcA*	Heat-inducible transcription repressor HrcA
gene0265	Contig2	*csp-1*	Cold shock-like protein
gene0776	Contig5	*cspD*	Cold shock protein CspD
gene0942	Contig6	*cspLA-1*	Cold shock-like protein CspLA
gene2106	Contig21	*cspLA-2*	Cold shock-like protein CspLA
gene2214	Contig25	*cspLA-3*	Cold shock-like protein CspLA
gene2237	Contig25	*csp-2*	Cold shock protein 1
gene1054	Contig6	*rnr*	Ribonuclease R
Lactate synthesis			
gene0302	Contig2	*ldhB*	L-lactate dehydrogenase 2
gene2269	Contig27	*ldh*	L-lactate dehydrogenase

**Table 2:** Putative antibiotic resistance genes in *E*. *faecium* YC07 genome.

Gene ID	Location	Gene Name	Antibiotics	Product	Identify (%)
gene1371	Contig10	*AAC(6′* *)-Ii*	aminoglycoside	chromosomal-encoded aminoglycoside acetyltransferase	98.9
gene1139	Contig7	*efmA*	fluoroquinolone; macrolide	MFS transporter permease	74.5

**Table 3:** Putative virulence factors in *E*. *faecium* YC07 genome.

Gene ID	Location	Gene Name	Predicted Functions	Identify (%)
gene0412	Contig3	*acm*	collagen adhesin precursor	95.5
gene2228	Contig25	*sgrA*	surface protein from Gram-positive cocci, anchor region	93.5
gene1872	Contig17	*bopD*	sugar-binding transcriptional regulator, LacI family	86.3
gene2420	Contig35	*bopD*	sugar-binding transcriptional regulator, LacI family	84.5
gene2112	Contig21	*clpP*	ATP-dependent Clp protease proteolytic subunit	81.1
gene1467	Contig11	*cpsA*	undecaprenyl diphosphate synthase	79.4
gene1519	Contig11	*bsh*	bile salt hydrolase	78.1
gene0411	Contig3	*acm*	collagen adhesin precursor	75.1
gene0412	Contig3	*acm*	collagen adhesin precursor	95.5

**Table 4:** The result of acute oral toxicity test.

Sex	Dose (mL/kg BW)	Test Animals (n)	Weight (X ± SD) (g)			Death of Animals (n)	Death Rate (%)
			0 Day	7 Day	14 Day		
Male	20.0	6	22.01 ± 0.09	22.02 ± 0.06	21.93 ± 0.09	0	0
Female	20.0	6	21.98 ± 0.14	21.99 ± 0.07	22.10 ± 0.11	0	0

## Data Availability

The original contributions presented in this study are included in the article/Supplementary Materials. Further inquiries can be directed to the corresponding author. The raw genome sequence of *Enterococcus faecium* YC07 has been deposited in the SRA of the NCBI under the accession number PRJNA1172820.

## Supplementary Materials

The following supporting information can be downloaded at: https://www.mdpi.com/article/10.3390/foods14122076/s1, Figure S1: Colonies of YC07 were grown on MRS agar plate (a). Gram staining of YC07, and the slide was visualized by light microscopy under a 1000X oil immersion objective (b); Figure S2: Analysis of GC-depth and K-mer frequency distribution of *E*. *faecium* YC07. GC-depth point diagram (a), K-mer frequency diagram (b); Figure S3: Bacteriostatic activity of *E*. *faecium* YC07. *S*. *aureus* on the left (a) and *E*. *coli* on the right (b). In the figure, a, b represent the two parallel experiments, and c represents the control experiment; Figure S4: In vitro safety assessment of *E*. *faecium* YC07. YC07 showed γ-hemolysis (a). The positive control *S*. *aureus* produced an obvious zone of β-hemolysis (b). Determination of the ability of YC07 to produce biogenic amine (c); Table S1: Genome of nine strains of the *Enterococcus* genus; Table S2: General properties and statistics of *E*. *faecium* YC07 genome.
