# Supplementary material for: From Isolation to Pilot-Scale Production: Enterococcus faecium YC07 with Urate-Lowering Potential from Fermented Food Jiangshui
Source: Foods. 2025 Jun 12;14(12):2076. doi: 10.3390/foods14122076 (PMC12192497; doi:10.3390/foods14122076)
Supplement: Supplementary file 1 [file foods-14-02076-s001.zip › foods-3634115-supplementary.pdf]

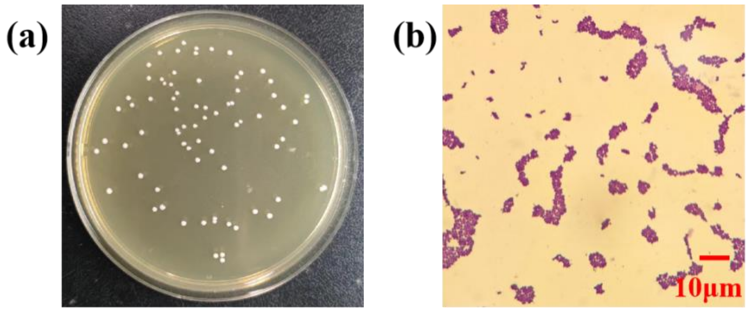

Figure S1. Colonies of YC07 were grown on MRS agar plate (a). Gram staining of YC07, and the slide was visualized by light microscopy under a 1000X oil immersion objective (b).

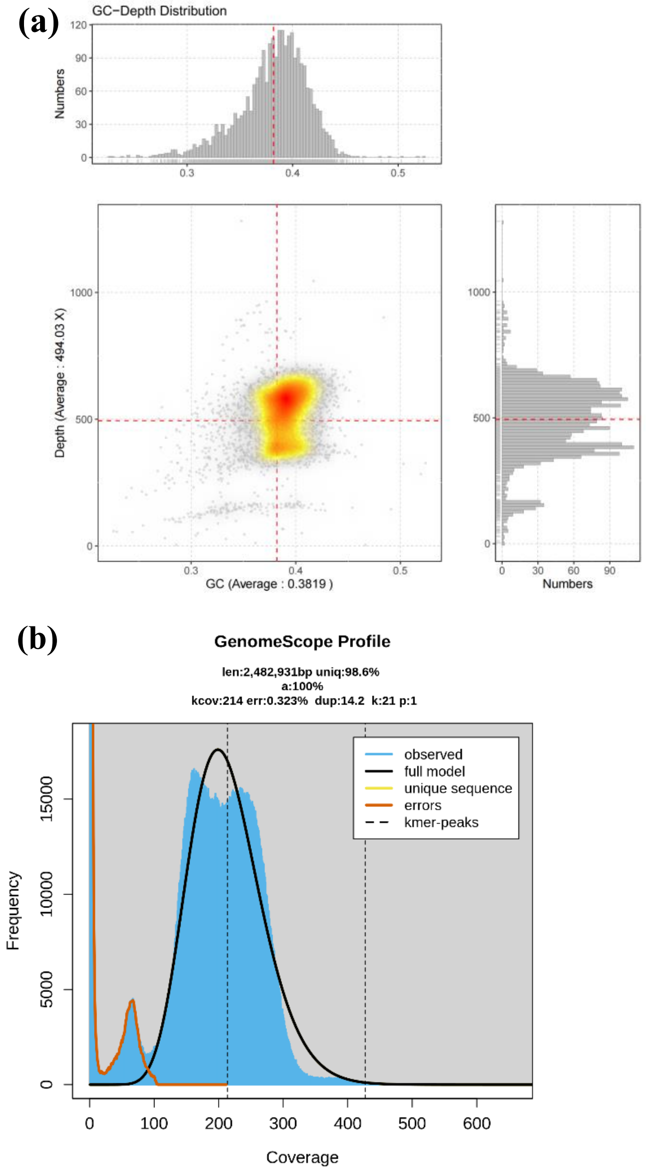

Figure S2. Analysis of GC-depth and K-mer frequency distribution of *E. faecium* YC07. GC-depth point diagram (a), K-mer frequency diagram (b).

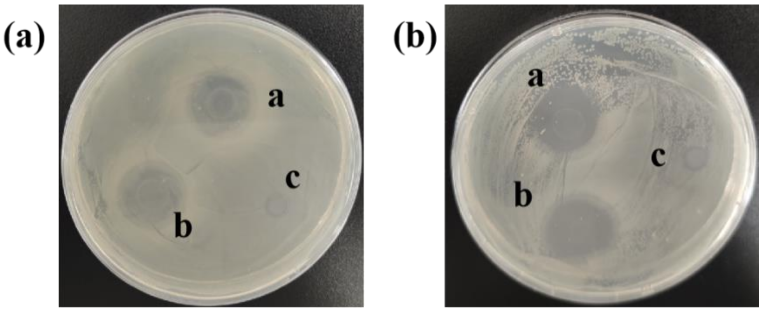

Figure S3. Bacteriostatic activity of *E. faecium* YC07. *S. aureus* on the left (a) and *E. coli* on the right (b). In the figure, a, b represents the two parallel experiments, and c represents the control experiment.

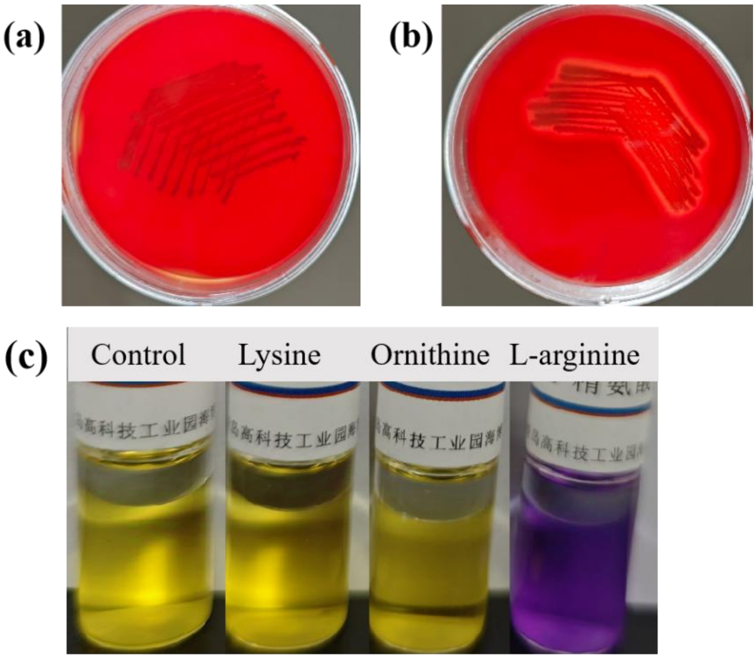

Figure S4. In vitro safety assessment of *E. faecium* YC07. YC07 showed γ-hemolysis (a). The positive control *S. aureus* produced an obvious zone of β-hemolysis (b). Determination of the ability of YC07 to produce biogenic amine (c).

Table S1. Genome of nine strains of the *Enterococcus* genus.

NCBI strain identifiers	GenBank accession	Genome size (Mb)	CDS
Enterococcus faecalis ATCC 19433	GCA_000392875.1	2.9	2761
Enterococcus faecalis T5	GCA_000393015.1	2.9	2680
Enterococcus faecium 6E6	GCA_001518735.1	3.4	3234
Enterococcus faecium ATCC 700221	GCA_001594345.1	3.2	2725
Enterococcus faecium UW8175	GCA_001587115.1	2.9	2669
Enterococcus faecium EFE10021	GCA_900066025.1	2.6	2487
Enterococcus faecium TK-P5D	CP045602.1	2.7	2676
Enterococcus lactis T110	GCA_000737555.1	2.7	2522
Enterococcus hirae ATCC 9790	GCA_000271405.2	2.9	2755

Table S2. General properties and statistics of *E. faecium* YC07 genome.

Features	Description
Clean reads bases	1308078066 bp
Q20 bases	1284422133 bp
Q30 bases	1246476012 bp
GC content	38.19%
Genome size	2.5 Mb
Contigs	59
N50	98733 bp
Total predicted genes	2448
CDS	2387
rRNA	6
tRNA	54
Pseudo-gene	0
ncRNA	1
